# *In-vitro* cytotoxic and pro-apoptotic effects of an *Echinococcus granulosus* antigen B nanoformulation on CT26 colorectal cancer cells

**DOI:** 10.3389/fonc.2026.1899949

**Published:** 2026-09-28

**Authors:** Soheil Sadr, Abbas Rahdar, Cinzia Santucciu, Nooshinmehr Soleymani, Hadi Mohebalian, Hassan Borji

**Affiliations:** 1 Department of Pathobiology, Faculty of Veterinary Medicine, Ferdowsi University of Mashhad, Mashhad, Iran; 2 Department of Physics, University of Zabol, Zabol, Iran; 3 WOAH and NRL for Echinococcosis, Animal Health, Istituto Zooprofilattico Sperimentale della Sardegna, Sassari, Italy

**Keywords:** apoptosis, colon cancer, *Echinococcus granulosus*, murine cell line, nanocapsules, nanotechnology, parasite

## Abstract

**Background/Objectives:**

In recent decades, many studies have examined the anticancer effects of parasites, but little attention has focused on enhancing these effects through nanotechnology, leaving a significant knowledge gap. Therefore, this study aims to enhance the *in-vitro* cytotoxic activity of *Echinococcus granulosus* Antigen B (AgB) against CT26 colorectal cancer cells using a nanoformulation.

**Methods:**

This study used the colon tumor 26 (CT26) and Human foreskin fibroblast-3 (HFF-3) cell lines. Cells were treated with free or nanocapsulated AgB at 0.25, 0.5, and 1.0 mg/mL for 24, 48, and 72 h. MTT assay was used to evaluate cell viability, and Annexin V/PI staining was performed to assess apoptosis.

**Results:**

The mean nanocapsule size was 180.35 nm, and the Polydispersity Index (PDI) was 0.2600. Scanning electron microscopy (SEM) confirmed that the nanocapsules were spherical. Cytotoxicity against CT26 cells increased with both concentration and incubation time for free and nanocapsulated AgB. Nanocapsulated AgB consistently showed higher cytotoxicity than free AgB at matched nominal formulation concentrations and time points. At 72 h, 1.0 mg/mL nanocapsulated AgB showed the highest cytotoxicity (28.6 ± 0.6%), followed by free AgB at the same concentration (18.7 ± 0.5%). In HFF3 cells, the maximum cytotoxicity was observed at 72 h with 1.0 mg/mL free AgB (4.7 ± 0.2%) and nanocapsulated AgB (3.7 ± 0.1%), which were lower than the corresponding values in CT26 cells.

**Conclusions:**

In conclusion, the AgB-loaded nanoformulation exhibited greater *in-vitro* cytotoxic and pro-apoptotic effects against CT26 colorectal cancer cells than free AgB, although these preliminary findings require further *in-vivo* validation before any translational conclusions can be drawn.

## Graphical Abstract

**Figure:**
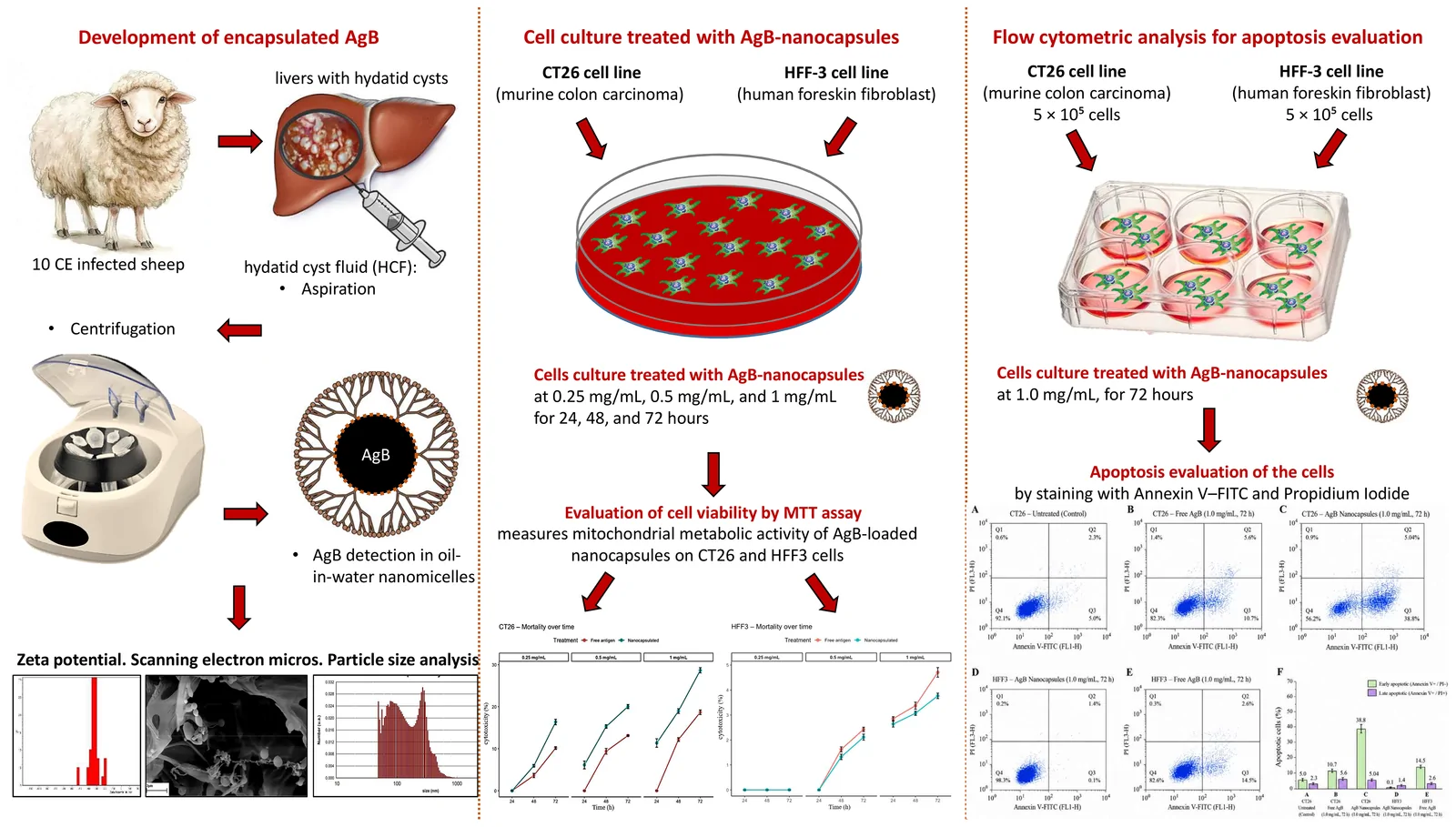

## 1 Introduction

Approximately 19.3 million new cancer cases were diagnosed worldwide in 2020, and nearly 10.0 million died each year, according to GLOBOCAN 2020 (1). Moreover, it has been estimated that 28.4 million new cancer cases will occur worldwide throughout the course of the 2040s (2). Immunotherapy has an advantage over traditional cancer treatments because it harnesses and manipulates the patient’s immune system to kill cancer cells (3). Cancer immunotherapy targets cancer antigens on malignant cells, alerts the immune system to foreign substances, and eradicates cancer through coordinated immune responses; normal cells without cancer antigens are not affected (4, 5).

Recent studies have reported that chronic parasite infections elicit anticancer innate immune responses, and parasite-based cancer immunotherapy has shown promising results in inhibiting cancer cell growth (6). Notably, several parasites, such as *Echinococcus granulosus* (*E. granulosus*), *Trichinella spiralis* (*T. spiralis*), and *Trypanosoma cruzi* (*T. cruzi*), have been shown to inhibit cancer cell proliferation (7–14). Tumor-associated antigens, such as Tn (GalNAc O-Ser/Thr), have been detected in the larval and adult forms of *E. granulosus*, as well as in circulating Tn antigen in patients with hydatid disease (15).

According to the hygiene theory, reduced exposure to microbial agents and parasites during childhood, due to improved hygiene conditions in modern societies, can increase the incidence of allergic and autoimmune diseases (16). Hygiene theory emphasizes the immunomodulatory role of parasites and suggests that balanced exposure to these agents can help regulate immune responses and prevent excessive immune activation against innocuous antigens or self-tissues (17). In this regard, antigen B derived from *E. granulosus* (EgAgB) is considered one of the most important immunomodulatory factors in *E. granulosus*. Structurally similar to vertebrate plasma lipoproteins, this molecule can transport neutral and polar lipids, such as triacylglycerides and sterols, thereby interfering with host lipid metabolism (18). AgB’s mechanism of action is complex and multifaceted; it modulates the host immune system directly by inhibiting polymorphonuclear cell chemotaxis and indirectly by altering the Th1/Th2 cytokine ratio and inducing a Th2-type response characteristic of chronic hydatidosis infection (19). This unique ability of AgB to suppress inflammatory immune responses and create an immune-tolerant environment has attracted researchers’ attention for its potential application in cancer therapy. Since cancerous tumors need to escape immune system surveillance for their growth and progression, the use of AgB as an immunomodulatory agent can open new horizons in cancer immunotherapy by suppressing chronic inflammation, which is one of the main factors of tumorigenesis, and also by changing the tumor microenvironment in favor of anti-cancer responses, and can be considered as a complementary strategy alongside conventional treatments (20, 21).

On the other hand, nanotechnology has shown significant advantages in programs aimed at combating parasitic infections (22–25). Because nanotechnology preserves the active ingredient more effectively and delivers it to the site of damage, the main advantages are improved therapeutic effectiveness and fewer adverse effects (26–28). In addition, nanoparticles have enabled the development of diagnostic procedures that are both more sensitive and faster, thereby improving the treatment of parasitic infections (29).

Nanotechnology and nanocapsules provide a significantly enhanced platform for drug delivery systems (DDS) (30–32). Nanotechnology and nanocapsules/nanovesicles have also significantly enhanced cancer delivery systems (33–35). The precision and efficiency offered by nanotechnology enable improved DDS, ensuring targeted and controlled release of therapeutic agents (36). Nanocapsules, with their small size and tailored properties, can cross biological barriers more effectively, enhancing drug bioavailability and therapeutic efficacy (37, 38). This approach holds great promise for advancing medical treatments, particularly in cancer therapy, where precise DDS targeting is crucial for therapeutic success (39, 40). Due to their small size and tunable surface area, nanocapsules can penetrate biological barriers, such as the blood-brain barrier, and effectively treat complex diseases, including cancer, neurological diseases, and drug-resistant infections (41). In addition, nanocapsules improve targeting effects (42, 43) and can improve efficacy by delivering drugs more precisely throughout the body (44).

In recent years, many studies have examined the anticancer effects of parasites and their antigens and evaluated the hygiene theory, but few have focused on enhancing these effects through nanotechnology, leaving a significant knowledge gap. Several studies have shown that parasite-derived antigens, particularly the *E. granulosus* B antigen, exhibit significant immunomodulatory and anticancer properties (45, 46). The parasite’s antigen shifts the tumor microenvironment toward a Th1 immune response by inducing mitochondrial pathway-dependent apoptosis, increasing the expression of proinflammatory factors such as Tumor Necrosis Factor-alpha (TNF-α) and Interferon-gamma (IFN-γ), and decreasing the expression of regulatory markers such as Interleukin-10 (IL-10) and Transforming Growth Factor-beta (TGF-β) (47, 48).

Many studies have examined the anticancer effects of parasites, but little attention has been paid to enhancing these effects through nanotechnology, leaving a significant knowledge gap. Hence, the present study aims to evaluate the *in-vitro* cytotoxic effects of free *Echinococcus granulosus* antigen B (AgB) and its nanoformulated preparation against CT26 colorectal cancer cells.

## 2 Materials and methods

### 2.1 AgB preparation

In July 2024, 10 sheep livers infected with hydatid cysts were collected immediately after slaughter from an industrial slaughterhouse in Mashhad. All samples were transported to the Veterinary Parasitology laboratory at Ferdowsi University of Mashhad under completely sterile conditions in sealed, insulated containers containing dry ice. Hydatid cyst fluid (HCF) was aspirated from the liver cysts using a sterile syringe after disinfection of the cyst surface with alcohol. The pooled HCF from all cysts was centrifuged at 1,500 g for 30 minutes to remove particulate debris.

To purify AgB, the supernatant was dialyzed against 0.005 M acetate buffer (pH 5.0) for 24 hours at 4 °C. The dialysate was then centrifuged at 30,000 g for 30 minutes at 4 °C to precipitate insoluble proteins, including AgB and Ag5. The precipitate was dissolved in 0.2 M phosphate buffer (pH 8.0), and globulins were removed by gradually adding ammonium sulfate powder to 40% saturation, followed by centrifugation at 3,000 g for 30 minutes. The supernatant was then heated in a water bath at 85 °C for 15 minutes to denature and precipitate the heat-labile Ag5 protein. After centrifugation at 30,000 g for 1 hour, the supernatant containing AgB was collected and filtered through a 0.2 µm filter (49).

To confirm the purity and molecular weight of the isolated AgB, SDS-PAGE was performed using a 12% separating gel and 5% stacking gel under reducing conditions. The gel was stained with Coomassie Brilliant Blue G-250. A single prominent band corresponding to a molecular weight of approximately 20 kDa was observed, consistent with the known molecular weight of *E. granulosus* AgB (11, 50). The final protein concentration was determined using the Bradford method with bovine serum albumin (BSA) as the standard, and the purified AgB was stored at −20 °C until use. A single batch of AgB was prepared and used for all experiments to ensure consistency. The species was identified as *Echinococcus granulosus* sensu lato based on the morphological characteristics of the hydatid cysts collected from sheep liver. Molecular confirmation of the species/genotype was not performed; therefore, the findings are attributed to *E. granulosus* sensu lato rather than to a specific genotype.

### 2.2 Development of encapsulated AgB

The AgB-loaded nanoformulation was prepared by dissolving AgB in 1% (w/w) ethyl butyrate oil with fatty acid sodium caprylate (SC, 0.09 g) and F127 (0.009 g) in phosphate-buffered saline (PBS, pH 7.4) under vigorous stirring at a fixed ethyl butyrate-to-surfactant molar ratio of 1 and a final total volume of 50 mL. Dialysis was performed for 24 h to remove excess free antigen (51, 52).

### 2.3 Zeta potential, scanning electron microscopy, and particle size analysis

Twenty-four hours before examination, the antigen solutions were prepared using AgB-loaded nanoformulation according to the instructions. Then, each prepared sample was poured into a separate microtube, mixed with 10 mL PBS, and transferred to the central laboratory. The particle size and size distribution of the nanocapsules obtained were evaluated with a particle size analyzer (PSA). Additionally, the measurements were reconfirmed at predetermined intervals. Zeta potential was determined using Zeta-Chek (Microtrac, ZC007, Germany). For scanning electron microscopy (SEM), samples were first prepared using the Sputter Coater (SC7620), Polaron Range, and then imaged on a LEO 1450VP.

### 2.4 Cell culture

The current study used the CT26 and HFF-3 cell lines, of animal and human origin, respectively, in accordance with current legislation and guidelines. The CT26 murine colon carcinoma cell line (RRID: CVCL_7254) and the HFF-3 human foreskin fibroblast cell line (RRID: CVCL_DB29) were obtained from the cell culture facility at Ferdowsi University of Mashhad, Mashhad, Iran, and the protocols used were approved by the Animal Welfare Committee at Ferdowsi University of Mashhad (Approval ID: 59513).

CT26 cells were cultured in RPMI-1640 medium supplemented with 10% fetal bovine serum (FBS), 100 U/mL penicillin, and 100 µg/mL streptomycin. HFF3 cells were cultured in Dulbecco’s Modified Eagle Medium (DMEM) supplemented with 10% FBS and antibiotics. Cells were maintained at 37 °C in a humidified atmosphere containing 5% CO_2_. The culture medium was refreshed every 48–72 hours to maintain adequate nutrient supply and remove metabolic waste products. Cells were passaged at approximately 80–90% confluence, typically every 2–3 days, depending on the growth rate. For passaging, the medium was removed, cells were washed with phosphate-buffered saline (PBS), detached using 0.25% trypsin-EDTA solution, and subcultured at a split ratio of 1:3 to 1:5. Cells from passages 5–15 were used for all experiments.

The HFF3 non-malignant comparator cell line group and CT26 cell line group were treated in triplicate with nanocapsules and free AgB at 24, 48, and 72 hours at concentrations of 0.25 mg/mL, 0.5 mg/mL, and 1 mg/mL.

### 2.5 Counting cells and trypan blue viability test

To ensure that the CT26 cells could survive the transfer into the Falcon, they were centrifuged. The mixture was then pipetted with 1 mL of sterile RPMI medium. In a microtube, 20 µL of the suspension was added along with Trypan Blue to observe the stained results. Under the microscope, 10 µL of the mixture were injected between Neubauer slides, and after that, the cells were counted just after injection.

### 2.6 Evaluation of cell viability by MTT assay

The cytotoxic effects of free AgB and AgB-loaded nanocapsules on CT26 and HFF3 cells were evaluated using the 3-[4,5-dimethylthiazol-2-yl]-2,5-diphenyl tetrazolium bromide (MTT) assay, which measures mitochondrial metabolic activity as an indicator of cell viability.

CT26 cells were harvested, counted, and seeded at a density of 1 × 10^4^ cells per well in 96-well flat-bottom plates using RPMI-1640 medium supplemented with 10% fetal bovine serum (FBS). After overnight incubation to allow cell attachment, the medium was replaced with fresh medium containing varying concentrations (0.25, 0.5, and 1.0 mg/mL) of either free AgB or AgB-loaded nanocapsules. Triplicate wells were prepared for each treatment condition. HFF3 cells were similarly treated under identical conditions as a non-malignant comparator cell line. For each experiment, the following control groups were included:

- Untreated control: cells cultured in medium alone (without any treatment) to establish baseline cell viability.
- Vehicle control: cells treated with DMSO at the same final concentration used for formazan dissolution, to rule out any solvent-related cytotoxicity.
- Cell-free blank: wells containing culture medium and the nanoformulation (at each tested concentration) without cells, to exclude possible optical interference or direct chemical reduction of MTT by the formulation components.

Plates were incubated at 37 °C in a 5% CO_2_ atmosphere for 24, 48, or 72 hours. At the end of each incubation period, 10 µL of MTT solution (5 mg/mL in phosphate-buffered saline) was added to each well, and the plates were incubated for an additional 4 hours at 37 °C to allow formazan crystal formation. Following incubation, the plates were centrifuged at 1,800 rpm for 10 minutes to pellet the crystals. The supernatant was carefully removed, and 150 µL of sterile DMSO was added to each well to dissolve the formazan crystals. The plates were then placed on a shaker for 10–15 minutes at room temperature to ensure complete dissolution.

The optical density (OD) of each well was measured at 570 nm using an ELISA microplate reader (BioTek, USA). For each sample, the background absorbance measured in the corresponding cell-free blank wells was subtracted from the sample readings. Cytotoxicity (%) was calculated using the following formula:

Cytotoxicity(%)=(1−OD570 of treated cells÷OD570 of untreated control cells)×100.

Both treated and untreated OD values were first corrected by subtracting the respective blank absorbance. Cytotoxicity was calculated separately for each time point (24, 48, and 72 h) relative to the corresponding time-matched untreated control cells incubated for the same duration. This approach ensured that normalization accounted for time-dependent changes in baseline cell viability. The MTT experiment was performed once, with three technical replicate wells per condition. These technical replicates were used to assess within-experiment variability and were not considered independent biological replicates.

### 2.7 Flow cytometry (annexin V and propidium iodide tests)

To evaluate the mode of cell death induced by free AgB and AgB-loaded nanocapsules, apoptosis was assessed using Annexin V-FITC and Propidium Iodide (PI) double staining. CT26 cells were seeded in 6-well plates at a density of 5 × 10^5^ cells per well and incubated overnight at 37 °C in 5% CO_2_. The cells were then treated with 1.0 mg/mL of either free AgB or AgB-loaded nanocapsules for 72 hours. This concentration was selected as the high-dose treatment concentration, representing the highest concentration tested in the MTT cytotoxicity assay.

For the non-malignant comparator cell line, HFF3 cells were treated under identical conditions with 1.0 mg/mL of free AgB or AgB-loaded nanocapsules for 72 hours.

After 72 hours of incubation, cells were harvested by trypsinization, washed twice with cold phosphate-buffered saline (PBS), and centrifuged at 1,500 rpm for 5 minutes. The cell pellet was resuspended in 195 µL of Annexin V binding buffer (1×) at a concentration of approximately 1 × 10^5^ cells per 100 µL. Subsequently, 5 µL of Annexin V-FITC and 5 µL of Propidium Iodide (PI) solution were added to the cell suspension according to the manufacturer’s instructions (BD Biosciences, USA). The mixture was gently vortexed and incubated for 15 minutes at room temperature in the dark. Following incubation, 400 µL of binding buffer was added to each tube, and the samples were analyzed immediately using a BD FACSCalibur flow cytometer (USA) equipped with a 488 nm argon laser.

For each sample, at least 10,000 events were acquired using CellQuest Pro software and analyzed using FlowJo software (v10.8, BD Biosciences). Cell populations were classified as:

- Viable cells: Annexin V^−^/PI^−^ (lower left quadrant).
- Early apoptotic cells: Annexin V^+^/PI^−^ (lower right quadrant).
- Late apoptotic/necrotic cells: Annexin V^+^/PI^+^ (upper right quadrant).
- Necrotic cells: Annexin V^−^/PI^+^ (upper left quadrant).

Quadrant gates were established using unstained cells and single-stained controls (Annexin V-FITC only and PI only) to ensure proper compensation and accurate population discrimination. The percentage of cells in each quadrant was recorded, and the results were expressed as mean ± SD from three technical replicates.

### 2.8 Statistical analysis

Statistical analyses were performed using R version 4.3.1. Data are presented as mean ± standard deviation (SD) of three technical replicate wells per condition from a single experiment. For each experimental condition, the three technical replicate wells were averaged to obtain one descriptive value. Because the experiment was not independently repeated, the technical replicates were not considered independent biological observations. The MTT findings are therefore presented as exploratory and descriptive observations and should be interpreted as preliminary evidence requiring confirmation in future experiments using independent biological replicates.

## 3 Results

### 3.1 Zeta potential, particle size analysis, and scanning electron microscopy

The zeta potential spectrum of the nanocapsules is shown in **Figure 1**. The nanocapsules exhibited a mean zeta potential of −33.46 mV (range: −18 to −55 mV). This negative zeta potential suggests a stable colloidal suspension with adequate electrostatic repulsion between particles. According to the PSA results, the mean nanocapsule size was 180.35 nm, and the polydispersity index (PDI) was 0.2600 (**Figure 2**). The SEM revealed that the nanocapsules exhibited a spherical morphology (**Figure 3**). This morphology is consistent with the expected nanoformulation structure.

**Figure 1:**
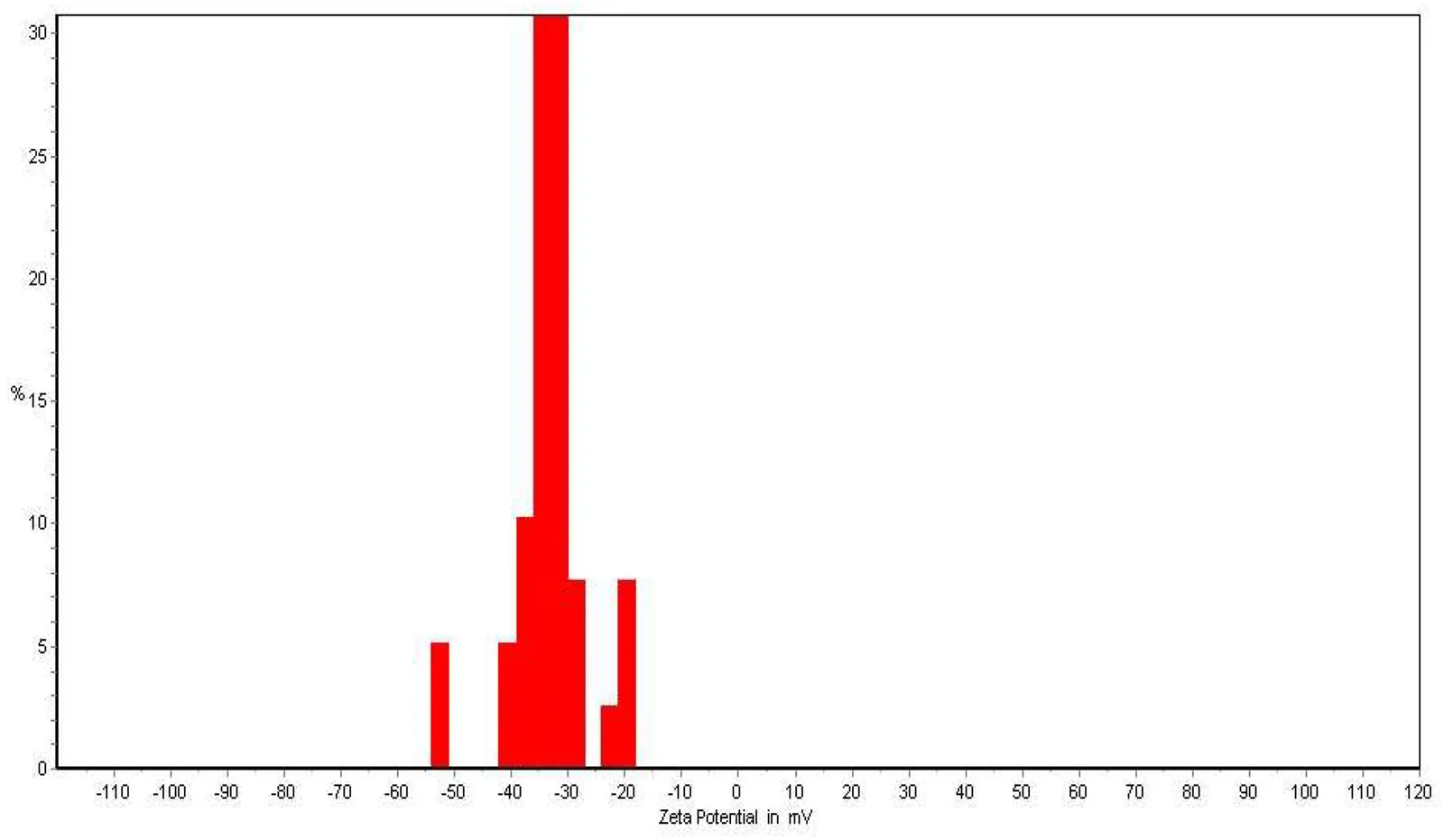
Zeta potential distribution of the AgB-loaded nanocapsules. The mean zeta potential was −33.46 mV (range: −18 to −55 mV), indicating a stable colloidal suspension.

**Figure 2:**
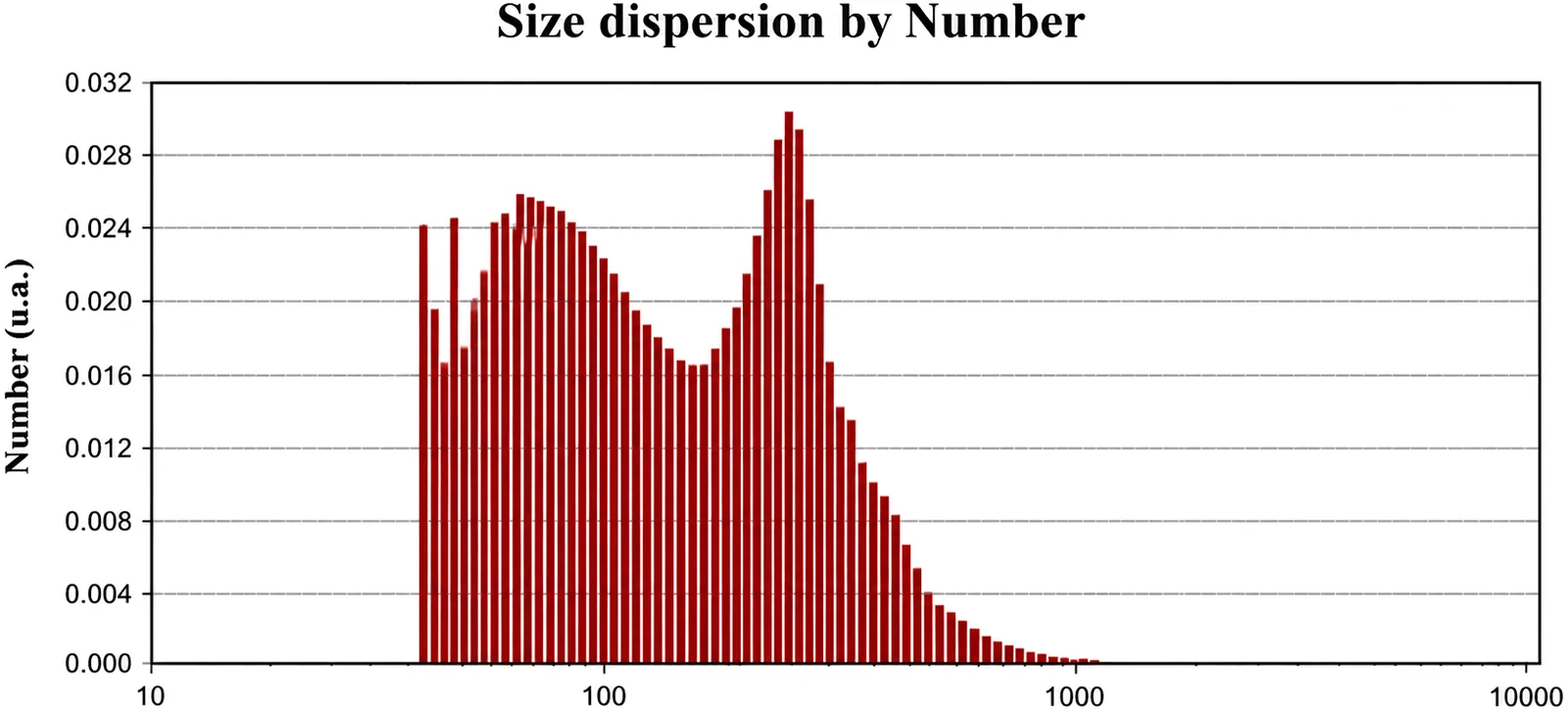
Particle size analysis showed a mean nanocapsule size of 180.35 nm and a polydispersity index of 0.2600.

**Figure 3:**
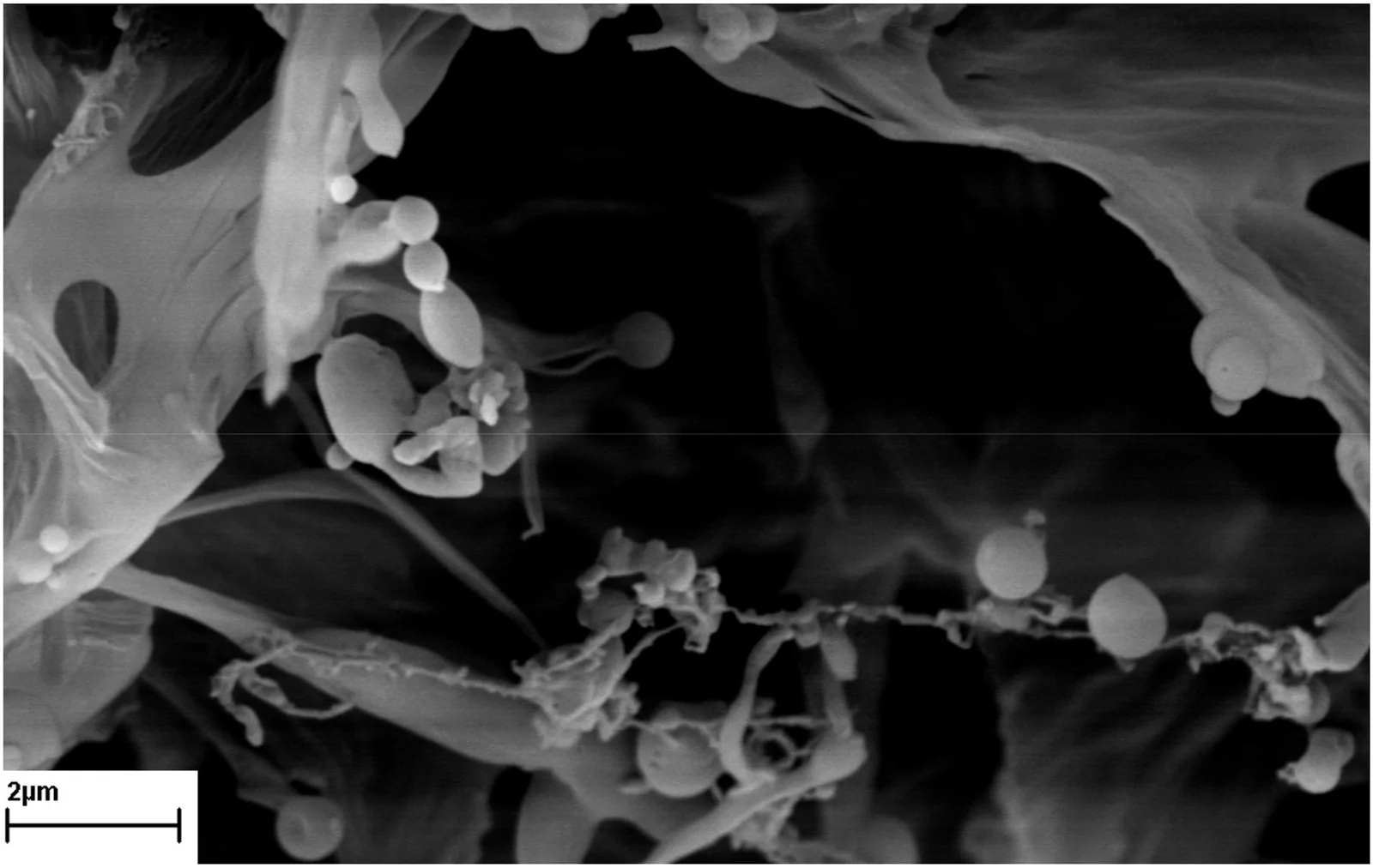
Scanning electron microscopy (SEM) image of AgB-loaded nanocapsules, showing spherical morphology.

### 3.2 Descriptive analysis of cytotoxicity

The MTT assay showed concentration- and time-dependent patterns of cytotoxicity in both CT26 and HFF3 cells. In CT26 cells, the nanocapsulated AgB formulation generally showed higher mean cytotoxicity than free AgB, particularly at higher concentrations and longer exposure times. In contrast, the overall cytotoxic response observed in HFF3 cells remained substantially lower across the tested conditions.

### 3.3 Cytotoxicity of CT26 at different concentrations and incubation times of free AgB and AgB nanocapsules

The descriptive MTT results indicated that CT26 cytotoxicity increased with increasing concentration and exposure time, with higher mean cytotoxicity generally observed for the nanocapsulated formulation than for free AgB. As shown in **Table 1**, free AgB and AgB nanocapsules showed concentration- and time-dependent cytotoxicity in CT26 cells. At 24 h, cytotoxicity remained minimal for all concentrations of free AgB, whereas nanocapsulated AgB at 0.5 and 1.0 mg/mL showed mean cytotoxicity values of 6.1 ± 0.5% and 10.9 ± 1.3%, respectively.

**Table 1:** Mean MTT-derived cytotoxicity (%) of CT26 cells treated with free and nanocapsulated AgB at different nominal concentrations and incubation times.

Concentration (mg/mL)	Time (h)	Free AgB	Nanocapsulated AgB	Control (Medium Alone, 72h)
0.25	24	≈0.0	≈0.0	1.2 ± 0.1
	48	3.6 ± 0.5	5.9 ± 0.3	
	72	10.1 ± 0.3	16.3 ± 0.6	
0.5	24	≈0.0	6.1 ± 0.5	
	48	9.4 ± 0.4	15.3 ± 0.4	
	72	13.1 ± 0.1	20.1 ± 0.5	
1.0	24	≈0.0	10.9 ± 1.3	
	48	12.1 ± 0.4	18.9 ± 0.5	
	72	18.7 ± 0.5	28.6 ± 0.6	

Data are presented as mean ± SD of three technical replicate wells per condition from a single experimental run. Cytotoxicity was calculated relative to the corresponding untreated control at each time point. The medium-alone value shown represents the baseline cytotoxicity observed at 72 h.

At 48 h, cytotoxicity increased with increasing concentration. Free AgB at 0.25, 0.5, and 1.0 mg/mL resulted in mean cytotoxicity values of 3.6 ± 0.5%, 9.4 ± 0.4%, and 12.1 ± 0.4%, respectively. At the same nominal concentrations, nanocapsulated AgB resulted in mean cytotoxicity values of 5.9 ± 0.3%, 15.3 ± 0.4%, and 18.9 ± 0.5%, respectively.

At 72 h, the highest observed cytotoxicity was obtained with 1.0 mg/mL nanocapsulated AgB (28.6 ± 0.6%), followed by 1.0 mg/mL free AgB (18.7 ± 0.5%). The medium-only control at 72 h showed 1.2 ± 0.1% cytotoxicity **Figure 4**.

**Figure 4:**
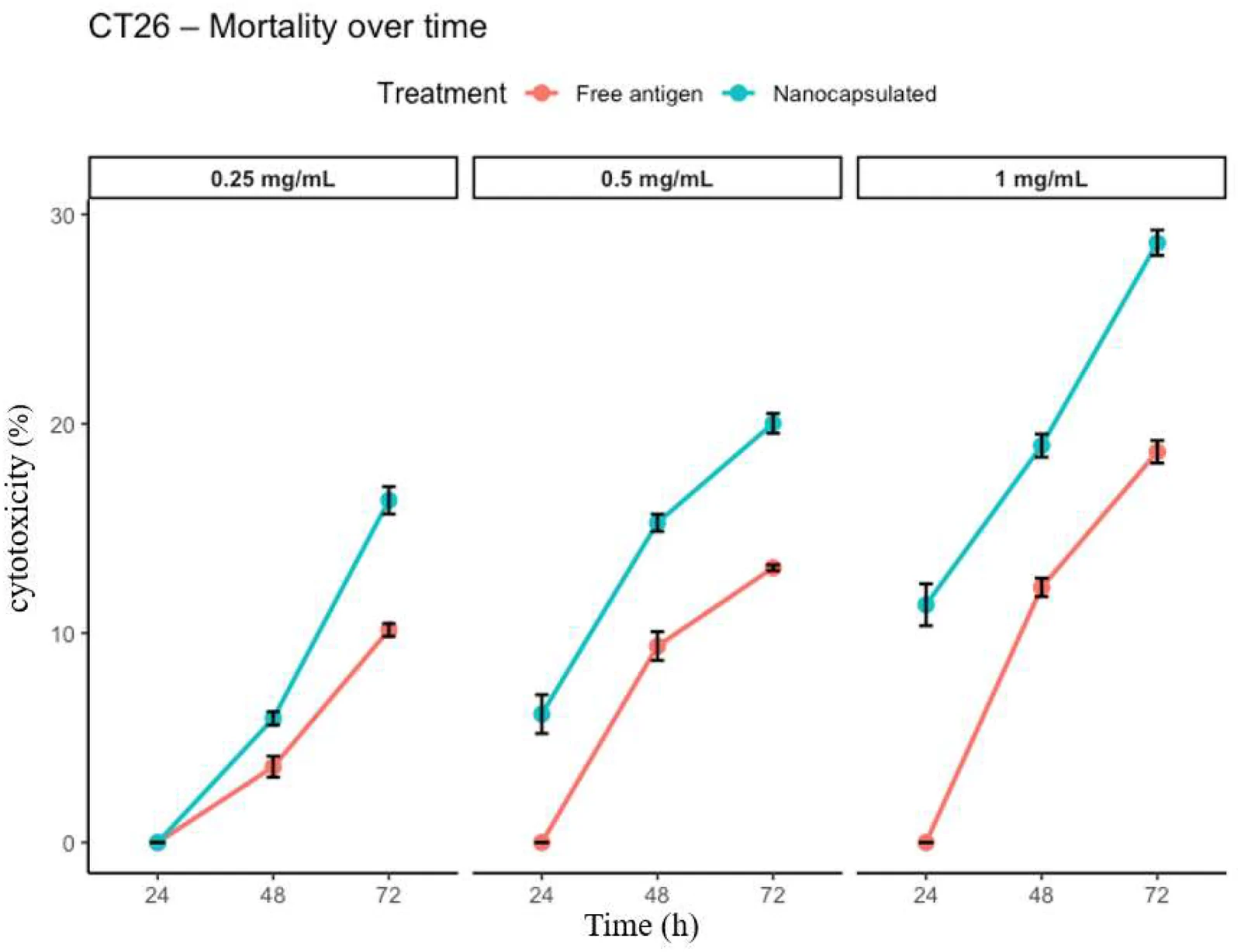
Time- and dose-dependent MTT-derived cytotoxicity of free and nanocapsulated AgB formulation on CT26 cells. CT26 cells were treated with free AgB or AgB-loaded nanocapsules at 0.25, 0.5, and 1.0 mg/mL for 24, 48, and 72 h. Data are presented as mean ± SD of three technical replicate wells per condition from a single experimental run. Individual technical replicate values are shown as scatter points. The figure illustrates the observed temporal and concentration-dependent patterns in cytotoxicity.

### 3.4 Cytotoxicity of HFF3 at different concentrations and incubation times of free AgB and AgB nanocapsules

Mean cytotoxicity in HFF3 cells was substantially lower than in CT26 cells across the tested concentrations and exposure times. The descriptive pattern suggests relatively limited cytotoxicity toward HFF3 cells under the tested *in-vitro* conditions. **Table 2** summarizes the cytotoxicity observed in non-cancerous HFF3 cells following treatment with free AgB and AgB nanocapsules. At 24 h, measurable cytotoxicity was observed only at the highest concentration (1.0 mg/mL), with mean values of 2.8 ± 0.1% for free AgB and 2.6 ± 0.2% for nanocapsulated AgB.

**Table 2:** Mean MTT-derived cytotoxicity (%) of HFF3 cells treated with free and nanocapsulated AgB at different nominal concentrations and incubation times.

Treatment concentration (mg/mL)	Time (h)	Free AgB	Nanocapsulated AgB	Control (Medium Alone, 72h)
0.25	24	≈0.0	≈0.0	0.3 ± 0.1
	48	≈0.0	≈0.0	
	72	0.5 ± 0.2	0.6 ± 0.8	
0.5	24	≈0.0	≈0.0	
	48	1.6 ± 0.1	1.3 ± 0.1	
	72	2.4 ± 0.2	2.1 ± 0.4	
1.0	24	2.8 ± 0.1	2.6 ± 0.2	
	48	3.3 ± 0.1	3.0 ± 0.2	
	72	4.7 ± 0.2	3.7 ± 0.1	

Data are presented as mean ± SD of three technical replicate wells per condition from a single experimental run. Cytotoxicity was calculated relative to the corresponding untreated control at each time point. The medium-alone value shown represents the baseline cytotoxicity observed at 72 h.

At 48 h, cytotoxicity remained low across the tested conditions. Free AgB at 0.5 and 1.0 mg/mL showed mean cytotoxicity values of 1.6 ± 0.1% and 3.3 ± 0.1%, respectively, whereas nanocapsulated AgB showed values of 1.3 ± 0.1% and 3.0 ± 0.2%, respectively.

At 72 h, the highest observed cytotoxicity was recorded at 1.0 mg/mL, with mean values of 4.7 ± 0.2% for free AgB and 3.7 ± 0.1% for nanocapsulated AgB. The medium-only control at 72 h showed 0.3 ± 0.1% cytotoxicity **Figure 5**.

**Figure 5:**
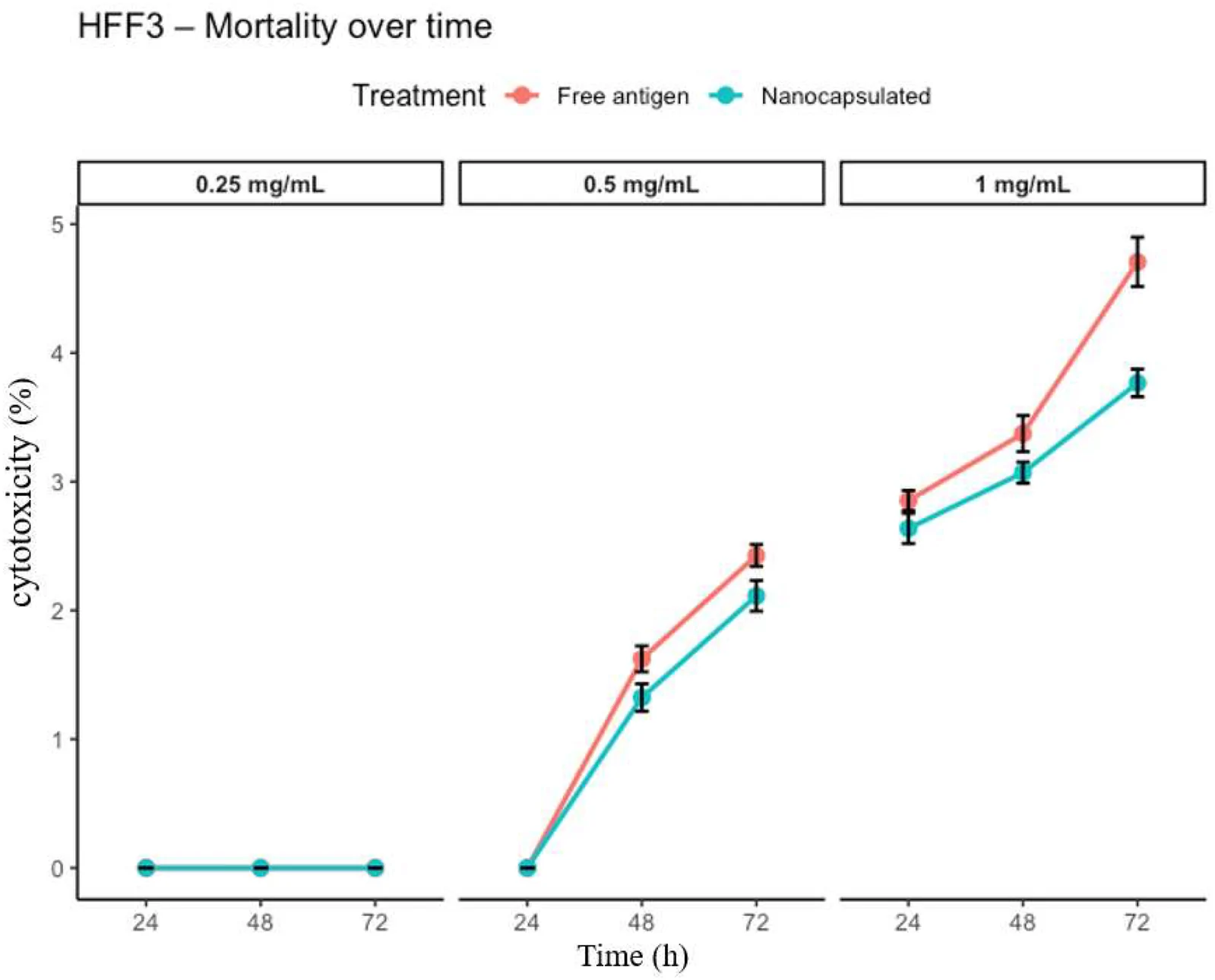
Time- and dose-dependent MTT-derived cytotoxicity of free and nanocapsulated AgB formulation on HFF3 cells. HFF3 cells were treated with free AgB or AgB-loaded nanocapsules at 0.25, 0.5, and 1.0 mg/mL for 24, 48, and 72 h. Data are presented as mean ± SD of three technical replicate wells per condition from a single experimental run. Individual technical replicate values are shown as scatter points. The figure illustrates the observed temporal and concentration-dependent patterns in cytotoxicity.

### 3.5 Flow cytometry and comparison of apoptosis rate

Flow cytometry results (**Figure 6**) showed that in CT26 cells, the untreated control group (**Figure 6A**) maintained a high percentage of viable cells (92.1%). Treatment with free AgB (**Figure 6B**) resulted in a decrease in viable cells (82.3%) with corresponding early apoptotic rates of 10.7% and late apoptotic rates of 5.6%. In contrast, treatment with AgB nanocapsules (**Figure 6C**) resulted in a marked decrease in viable cells (56.2%) and a marked increase in early (38.8%) and late (5.04%) apoptotic cells, indicating a stronger pro-apoptotic effect of the nanoformulated preparation. In HFF3 cells, both free AgB (**Figure 6E**) and AgB nanocapsules (**Figure 6D**) maintained high percentages of viable cells (82.6% and 98.3%, respectively) with low rates of apoptosis, suggesting a lower cytotoxic effect on this non-malignant cell line under the tested *in-vitro* conditions. Quantitative analysis of apoptotic populations is presented as bar graphs showing the percentages of early and late apoptotic cells (**Figure 6F**).

**Figure 6:**
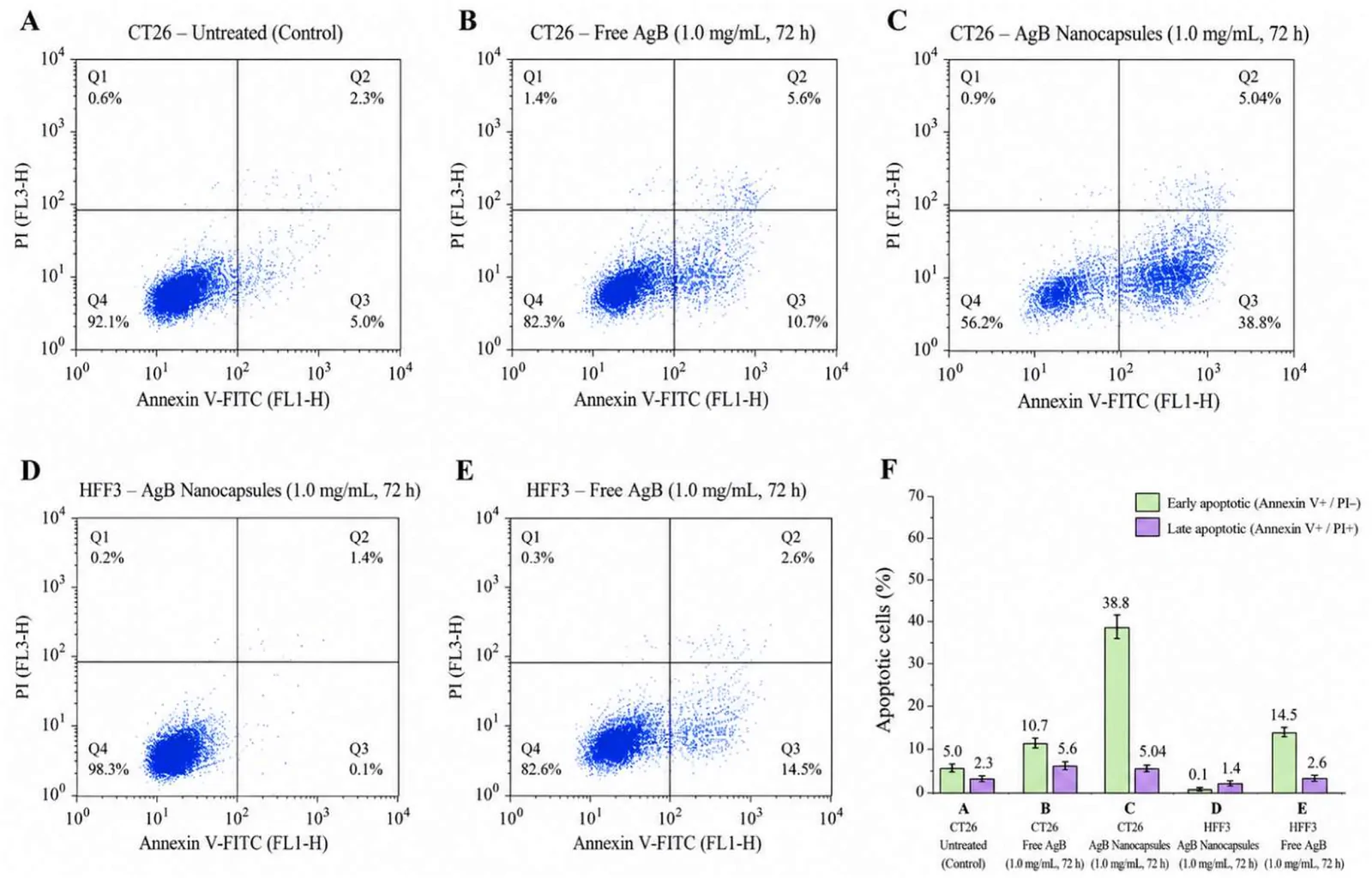
Flow cytometric analysis of apoptosis in different treatment groups. Cells were stained with Annexin V–FITC and Propidium Iodide and analyzed by flow cytometry. Representative dot plots **(A–E)** illustrate the distribution of viable (Annexin V^−^/PI^−^), early apoptotic (Annexin V^+^/PI^−^), late apoptotic/necrotic (Annexin V^+^/PI^+^), and necrotic (Annexin V^−^/PI^+^) cell populations. **(A)** Untreated CT26 cells (control); **(B)** CT26 cells treated with free AgB (1.0 mg/mL, 72 h); **(C)** CT26 cells treated with AgB nanocapsules (1.0 mg/mL, 72 h); **(D)** HFF3 cells treated with AgB nanocapsules (1.0 mg/mL, 72 h); and **(E)** HFF3 cells treated with free AgB (1.0 mg/mL, 72 h). Quantitative analysis of apoptotic populations is presented as bar graphs showing the percentage of early and late apoptotic cells **(F)**.

## 4 Discussion

Parasite-derived antigens have been investigated for their potential anticancer properties, with some studies reporting direct cytotoxic effects on tumor cells and immunomodulatory activities (53, 54). The current study investigates the inhibitory effect of nanoencapsulated *E. granulosus* antigen B on the growth of colon cancer cells (CT26) *in-vitro*. However, the present study is limited to *in-vitro* assessment of direct cell viability and apoptosis, and does not involve immune cells or *in-vivo* immune responses. Therefore, the observed effects should be attributed to direct cytotoxicity rather than to immunotherapy or immune system activation. Future studies incorporating immune-competent animal models would be required to evaluate any potential immunomodulatory contributions.

Immunotherapy uses the immune system’s power to target cancer cells (55). Since cancer cells may also express antigens similar to those found in parasite cells, these antigens have been used as therapeutic targets in cancer immunotherapy (56–58). This novel approach, based on evolved interactions between the parasite and the host immune system, shows that some parasites can modulate immune responses in favor of an antitumor environment (59). Recent studies suggest that antigens derived from parasites such as *Toxoplasma gondii* (60), *Trypanosoma cruzi*, and some helminths can alter the tumor microenvironment in favor of the immune system by stimulating Th1 responses and increasing the activity of natural killer cells and macrophages (61–65). In addition, certain parasite proteins can inhibit tumor angiogenesis and induce apoptosis in cancer cells (66, 67).

Nanotechnology and its application in cancer treatment have been recognized as an advanced field in medical science (68). One important method in this field is using nanocapsules as drug carriers to deliver drugs effectively to cancer cells (69). Because of their nanometer size, these particles are more likely to penetrate deeper tissues and cancer cells (70). Using nanocapsules in DDS offers advantages such as increased drug solubility and therapeutic efficacy and reduced accumulation of unstable drugs in the body, thereby enhancing cancer treatment effectiveness (71, 72). Additionally, nanocapsules can prevent drug accumulation in healthy cells, thereby minimizing unwanted side effects (73, 74).

Consistent with the current study’s findings, human HCF has been shown to exert cytotoxic effects on the immune system in an animal model of colon cancer (75). Anti-HCF antibodies can detect both surface and intracellular antigens in CT26 cells. In experiments aimed at preventing tumor development, HCF-containing vaccines protected 40% of mice from developing tumors. After vaccination, the remaining mice were protected from tumor re-challenge by a memory immune response that also led to adaptive immune responses, indicating that this response had been established. Anti-HCF antibodies were used to examine the CT26 cell proteome, revealing two antibody-recognized proteins: mortalin and M-type creatine kinase. Interestingly, mortalin in CT26 has 60% homology with hsp70 from *E. granulosus*. Additionally, a previous study investigated the effects of vaccination with *E. granulosus* antigens from human HCF on lung cancer development (9). Results showed that vaccination, as both a preventive and therapeutic measure, inhibits tumor growth and increases mouse survival. Vaccination with this fluid induces IgG antibodies that recognize LL/2 tumor cells and elicits an atypical immune response, with production of IL-5 and, to a lesser extent, IFNγ in tumor-draining lymph nodes and the spleen.

Moreover, a fraction of HCF was shown to effectively inhibit colon cancer tumor growth in BALB/c mice (76). Researchers used mouse colon cancer cells to challenge six groups of mice. Treatment groups received hydatid cyst fluid, the 78 kDa fraction, and live protoscolices after 5 days of tumor growth. Statistically significant differences in tumor sizes were observed between the study and control groups when mice were injected with hydatid cyst antigens, especially a fraction of cyst fluid. Furthermore, in a similar study, sera from rabbits immunized against hydatid cyst wall (HCW) antigens were incubated with 4T1 mammary carcinoma cells to investigate cross-reactivity between HCW antigen antisera and the surface antigens of breast cancer cells (10). Western blot analysis of breast cancer patient serum showed a significant band corresponding to HCW antigens on SDS-PAGE. Then, the protein bands were used to correlate with breast cancer serum for preventive immunization of BALB/C mice. HCW antigens significantly inhibited the growth of 4T1 breast tumors.

In our previous study, we investigated the antitumor effect of somatic antigens from hydatid cyst protoscoleces using K562 cancer cells (77). We examined the antitumor effects of protoscoleces antigens on K562 cancer cells, and the antigens were added for 24, 48, and 72 hours at 0.1 mg/ml, 1 mg/mL, and 2 mg/mL, respectively. The results showed that all three concentrations of protoscoleces hydatid cyst antigen significantly reduced cancer cell growth. In addition, the number of cancer cells undergoing apoptosis increased with increasing antigen exposure time. Flow cytometry results, however, showed increased apoptosis after treatment, consistent with the present study.

Moreover, the effect of HCF on causing apoptosis in the mouse breast cancer cell line has been investigated (78). Hydatid cyst fluid antigens were prepared, including AgB, glycolipid, glycoprotein, and the 78 kDa fraction. The results showed that the 78 kDa and glycoprotein fractions induced more than 40% apoptosis. Furthermore, HCF and glycolipid antigens induced 39% and 34% apoptosis, respectively, whereas the AgB fraction showed a lesser effect. Furthermore, the effect of HCF on the colon cancer cell line was investigated, and the results showed that the apoptosis rate increased (79). This antigen exhibited antitumor activity by increasing BCL2-associated X protein (BAX) expression and decreasing B-cell lymphoma 2 (BCL2) expression. Cell cycle analysis also showed that in the antigen-affected group, more cells were in the G0/G1 and G2/M phases, and fewer were in the S phase. Furthermore, another study showed that hydatid cyst antigens had therapeutic effects in C57/black mice with melanoma (80). Based on these results, tumor growth was inhibited in the studied group, and the average tumor sizes of the treated and control groups differed significantly.

The current study demonstrates cytotoxic effects of AgB and shows that the nanoformulated preparation produced a greater antitumor effect than free AgB under the tested conditions. The enhanced effect observed with the nanoformulation may be attributed to improved cellular uptake or altered bioavailability, although further studies are required to confirm the underlying mechanisms. It is suggested that further studies be expanded in the *in-vivo* setting. Future studies could include examining long-term effects, distribution across different sizes, and systemic safety. Moreover, improvements in nanotechnology techniques and methods are needed to increase the accuracy of AgB delivery to tumor cells and reduce potential side effects in the host. This future research will enable the development of optimal tumor treatment strategies using hydatid cyst-derived antigens.

Several limitations should be considered in the present study and kept in mind for future investigations. First, the loading capacity and encapsulation efficiency of AgB within the nanoformulation were not quantitatively determined after dialysis. Therefore, the exact AgB content retained in the nanoformulation and complete dose equivalence between free and nanoformulated AgB cannot be established from the available data. Accordingly, interpret the observed differences between the two formulations under the tested nominal concentrations rather than as definitive evidence of quantitatively enhanced AgB potency. Future studies should directly quantify AgB loading and encapsulation efficiency to establish precise dose equivalence and improve formulation characterization.

Another limitation of the present study is that the nanoformulation was evaluated exclusively under *in-vitro* conditions, and no specific route of administration was investigated. Consequently, its gastrointestinal stability, gastric-acid resistance, intravenous suitability, systemic biodistribution, pharmacokinetics, and *in-vivo* safety remain unknown. Although particle-size analysis showed a mean particle size of 180.35 nm, these physicochemical findings alone cannot establish suitability for oral or intravenous administration. Future *in-vivo* studies should therefore determine the appropriate administration route and comprehensively evaluate stability, biodistribution, pharmacokinetics, and safety before further translational development.

The present study also has limitations regarding the stability and safety characterization of the nanoformulation. Although basic physicochemical properties were characterized, dedicated long-term stability, physiological stability, and comprehensive *in-vivo* safety assessments were not performed. Future studies should address these aspects to establish the formulation’s robustness, safety, and translational potential. Additionally, independent cell-line authentication and mycoplasma testing were not performed. While the cell lines were obtained from a reputable source, future studies should include these quality-control measures to confirm cell-line identity and exclude mycoplasma contamination.

It should be acknowledged that an empty-carrier control (nanocapsules without AgB) was not included in the present study. Therefore, the possibility that the carrier components themselves contributed to the observed reduction in CT26 cell viability cannot be completely excluded. Accordingly, we interpret the results as indicating that the AgB-loaded formulation produced a greater *in-vitro* effect than free AgB under the tested conditions, rather than attributing the enhanced effect solely to AgB encapsulation. Future studies incorporating appropriate empty-carrier controls will be necessary to confirm that the observed cytotoxicity is specifically mediated by AgB.

Furthermore, while SEM confirmed spherical morphology and zeta potential analysis suggested colloidal stability, these physicochemical characterizations do not by themselves confirm AgB encapsulation or drug stabilization. To fully validate the nanoformulation, direct quantification of AgB loading capacity and encapsulation efficiency, as well as stability studies under physiological conditions, are required.

## 5 Conclusions

In conclusion, free *E. granulosus* AgB and its nanoformulated preparation showed concentration- and time-dependent patterns of cytotoxicity against murine CT26 colorectal cancer cells under the tested *in-vitro* conditions. At 1.0 mg/mL after 72 h, the AgB-loaded nanoformulation also showed a more pronounced pro-apoptotic effect than free AgB. The AgB-loaded nanocapsules produced a greater reduction in CT26 cell viability and a more pronounced apoptotic response than free AgB at the same nominal concentrations, while showing lower cytotoxicity in non-malignant HFF3 cells. The current study should be considered an exploratory proof-of-concept investigation, and future studies incorporating appropriate formulation characterization, *in-vivo* tumor models, and comprehensive safety assessments are necessary to validate the translational potential of this nanoformulation for anticancer applications.

## Data Availability

The raw data supporting the conclusions of this article will be made available by the authors, without undue reservation.
